# The association of post-discharge adverse events with timely follow-up visits after hospital discharge

**DOI:** 10.1371/journal.pone.0182669

**Published:** 2017-08-10

**Authors:** Dennis Tsilimingras, Samiran Ghosh, Ashley Duke, Liying Zhang, Henry Carretta, Jeffrey Schnipper

**Affiliations:** 1 Department of Family Medicine & Public Health Sciences, Wayne State University School of Medicine, Detroit, Michigan, United States of America; 2 Tallahassee Memorial Hospital, Tallahassee, Florida, United States of America; 3 Department of Behavioral Sciences and Social Medicine, Florida State University College of Medicine, Tallahassee, Florida, United States of America; 4 Division of General Medicine, Brigham and Women’s Hospital and Harvard Medical School, Boston, Massachusetts, United States of America; Stanford University School of Medicine, UNITED STATES

## Abstract

**Objective:**

There has been little research to examine the association of post-discharge adverse events (AEs) with timely follow-up visits after hospital discharge. We aimed to examine whether having a timely follow-up outpatient visit would reduce the risk for post-discharge AEs.

**Methods:**

This was a methods study of patients at risk for post-discharge AEs from December 2011 through October 2012. Five hundred and forty-five patients who were under the care of hospitalist physicians and were discharged home from a community hospital, spoke English, and could be contacted after discharge were evaluated. The aim of the study was to examine the association of post-discharge AEs with timely follow-up visits after hospital discharge based on structured telephone interviews, health record review, and adjudication by two blinded, trained physicians using a previously established methodology.

**Results:**

We observed a higher incidence of AEs with patients that had their first follow-up visit within 7 days after hospital discharge (33.5% vs. 23.0%, p = 0.007). This effect was attenuated somewhat but remained significant when adjusted for several patient factors (adjusted OR 1.33, 95% confidence interval 1.16–2.71).

**Conclusion:**

This observational study paradoxically showed an increase in post-discharge AEs with early follow-up, likely a result of confounding by indication and/or information bias that could not be completely adjusted for. This study illustrates the potential hazards with conducting observational studies to determine the efficacy of various transitional care interventions, such as early follow-up, where risk for confounding by indication is high.

## Introduction

Several studies have examined timeliness of outpatient follow-up visits with a goal of decreasing hospital readmissions [1–4]. These studies have specifically focused on targeting high risk patients [1], evaluating characteristics and outcomes of discharged patients lacking timely follow-up visits [2], targeting patients with follow-up visits within 7 days [3], and evaluating the relationship between outpatient follow-up appointments made and 30-day unplanned readmissions [4]. However, there is little information regarding the association of timely post-discharge follow-up outpatient visits with patients experiencing post-discharge adverse events (AEs), an outcome that is very prevalent, unpleasant to patients, burdensome to caregivers, expensive for the health care system, and may be easier to prevent or ameliorate than readmissions.

Post-discharge AEs are the injury that results from the care that is provided by health care professional approximately a month after discharge from the hospital [5]. As described in detail in previous studies, post-discharge AEs have become a major public health concern in the urban and rural adult population [6–8]. The outcomes that are associated with post-discharge AEs are staggering as described in the literature [6–8]. Therefore, we were compelled to investigate timely follow-up visits and post-discharge AEs a month after discharge from the hospital. Specifically, the objective of this study was to examine whether having a timely follow-up outpatient visit sooner rather than later would reduce the risk for post-discharge AEs.

## Methods

### Setting, participants, and study recruitment

This evaluation was conducted as part of a study evaluating post-discharge AEs in urban and rural patients discharged from an academically affiliated community hospital with a large proportion of both types of patients. The methods and results of that study have been previously reported [8,9]. Briefly, eligible subjects for this methods study were recruited from Tallahassee Memorial Hospital (TMH) from 14 December 2011, through 8 October 2012. Two study nurses approached adult patients and informed them of the study. If patients agreed to participate in the study, they received a written consent from the patients. We recruited adults admitted to the medical service, under the care of TMH hospitalist physicians, who were being discharged home, spoke English, and who could be contacted 30 days after discharge for a telephone interview. In the event that patients were unable to complete the telephone interview themselves, patient surrogates were permitted to complete the telephone interview [8]. Prior to discharge, nurse-reviewers obtained a release to allow researchers to review health records from other institutions in the month after discharge, and then administered a brief demographic survey regarding exposure variables difficult to obtain from health records, including education level, household income and living arrangements, transportation, and caregiver status. The study was approved by FSU, TMH, and Wayne State University institutional review boards.

### Telephone interviews

Nurse-reviewers made their first attempt to contact study patients by telephone within 3–4 weeks of discharge. If nurse-reviewers were unable to reach patients after ten attempts or within 6 weeks after discharge from the hospital, these patients were recorded as non-responders, and efforts to gather post-discharge health records were initiated, including healthcare utilization from TMH electronic data sources and review of local newspapers for obituaries and the State of Florida Vital Statistics registry to assist in the identification of deceased patients. The 20-minute telephone interviews included questions to determine a patient’s use of health services since discharge, both inside and outside the TMH system, including all outpatient follow-up visits after discharge, and a full review of organ systems (S1 Table) [8,10]. If patients answered that any of these symptoms were new or worse since discharge, the nurse-reviewer asked additional follow-up questions regarding the severity of the symptoms, the timing of symptoms in relation to hospitalization and treatments, and the resolution of symptoms, in order to determine the relationship between these symptoms and health care delivery. The 20-minute telephone interview has been utilized successful in two other similar studies with post-discharge AEs [6,7].

### Health records reviews

Nurse-reviewers combined information obtained from the telephone interview and/or the outpatient health records to screen for: 1) new or worsening symptoms; 2) unplanned health services utilization; and 3) abnormal laboratory test results. If nurse-reviewers identified any of the above information, they referred these cases to physician adjudicators who independently reviewed all information prepared by nurse-reviewers to determine the occurrence of postdischarge AEs. Two physician-adjudicators independently created case summaries for patients they identified with possible post-discharge AEs [6–8,11–13]. For each possible AE, the same physician-adjudicators then rated their confidence that the patient injury was a result of medical management and not the patient’s underlying medical conditions, including the absence of needed treatment when clearly clinically indicated [6–8,11–13], utilizing a scale of 1 to 6 [6–8,11–15]. As with previous studies using this scale if their rating was 4, 5 or 6, the event was considered an AE.

### Statistical analysis

Bivariate analyses were utilized to test the association between the time to first post-discharge follow-up and the incidence of post-discharge AEs in the overall sample. To avoid reverse causality (e.g., scheduling a prompt follow-up appointment because an adverse event had already occurred), we restricted the evaluation to follow-up visits that had been scheduled prior to hospital discharge. Multiple logistic regression models were then built to adjust for potential confounders of the relationship between timing of follow-up and the presence of any post-discharge AEs in each patient. We utilized SAS version 9.4 for all analyses (SAS Institute, Inc., Cary, North Carolina).

## Results

**Fig 1:**
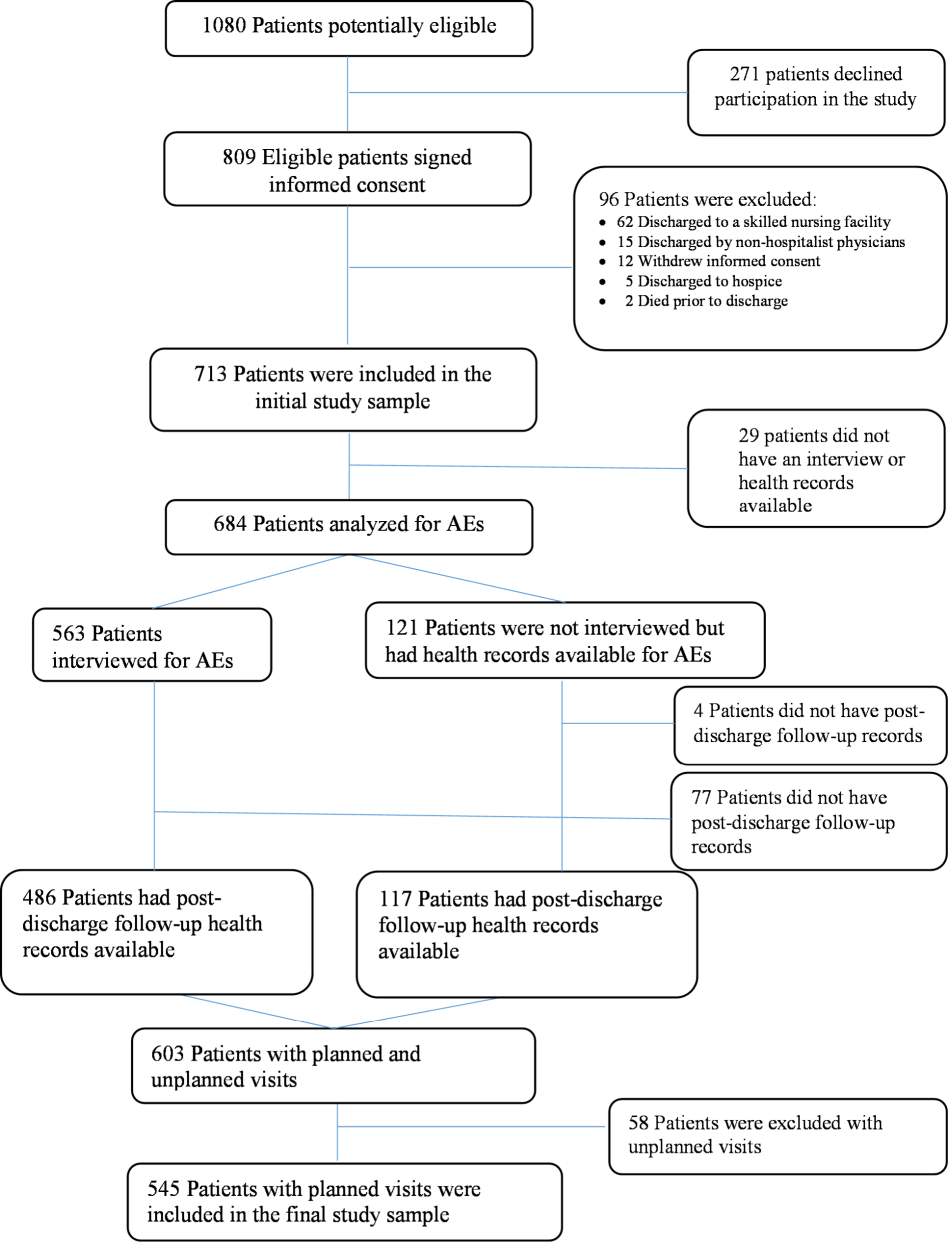
Flow of participants through the study. AEs = adverse events.

We identified 809 eligible patients who agreed to participate in the study (Fig 1). We excluded 96 patients because they were discharged to skilled nursing facilities or by non-hospitalist physicians, withdrew consent, or were discharged to hospice or died prior to discharge, and 29 patients were lost to follow-up. We also excluded 81 patients without post-discharge follow-up health records and 58 patients with unplanned outpatient visits. Finally, we have provided characteristics for the 271 and 168 patients that were excluded from the study (S2 Table).

Outcomes were assessed for 545 patients, with mean age of 62 years and a range of 22–93 years, 51% of whom were female, 44% with high school education or less, 59% with a median household income less than $50,000, 17% living alone, 52% with Medicare insurance, and 51% urban (Table 1). Hypertension, diabetes mellitus type 2, and coronary artery disease were among the most common diagnoses, the mean Diagnosis Related Group (DRG) weight was 0.08, and the mean Elixhauser comorbidity score was 2.25.

**Table 1:** Patient characteristics.

Characteristic	n (%)
Sex	
Female	278 (51.01)
Male	267 (48.99)
Race	
White	428 (78.50)
Non-White	117 (21.50)
Education	
Less than high school	56 (10.28)
High school	184 (33.76)
Some college	171 (31.38)
College degree	74 (13.58)
Post-graduate	60 (11.01)
Household income	
Less than $9,000	52 (10.90)
$9000–24,999	111 (23.27)
$25,000–49,999	120 (25.16)
$50,000–74,999	97 (20.34)
$75,000–99,999	56 11.74)
$100,000 or more	41 (8.60)
Missing	68 (12.47)
Living situation	
Lives alone	93 (17.06)
Does not live alone	452 (82.94)
Insurance type	
Private	204 (37.43)
Medicare	285 (52.29)
Medicaid	38 (6.97)
Self-pay	18 (3.30)
Living location	
Urban	279 (51.20)
Rural	266 (48.80)
Hypertension	391 (71.70)
Type 2 Diabetes Mellitus	188 (34.50)
Coronary artery disease	169(31.00)
Total Elixhauser comorbidity score, mean (SD)	2.25 (1.4)
Log (DRG weight), mean (SD)	0.08 (0.511)
Age, mean (SD)	61.7 (14.73)
Length of stay (per day), mean (SD)	3.78 (2.99)

*Note*: DRG = Diagnosis Related Group

In the bivariate chi-square analysis (Table 2), we observed a higher incidence of AEs when patients had their first scheduled post-discharge follow-up outpatient visit within 7 days (33.5%) than patients with visits of greater than 7 days (23%), p = 0.007.

**Table 2:** Unadjusted association between post-discharge adverse events and timely post-discharge follow-up visits (N = 545).

Follow-up visit time category	Number of patients in follow-up visit time category	Number of patients in follow-up visit time category experiencing AEs	Percent of patients in follow-up visit time category experiencing AEs (%)
0–7 days	319	107	33.5
More than 7 days	226	52	23.0
Total number of patients	545	159	29.2

*Note*: AEs = Adverse Events.

In the multiple logistic regression model, we included potential confounders such as patient’s age, living alone, insurance, and the severity of the patient’s primary diagnosis (DRG weight) and comorbidities (Elixhauser Score) (Table 3). The positive relationship between early planned follow up and post-discharge AEs was maintained, although the effect was attenuated somewhat (adjusted OR 1.33, 95% CI 1.16–2.71), p = 0.008).

**Table 3:** Adjusted effect of early planned follow-up and post-discharge adverse events. (N = 545)*.

Characteristic	Adjusted Odd Ratio (95% Confidence Interval)	P value
Scheduled Follow-Up Appointment within 7 days of discharge	1.33 (1.16–2.71)	0.008
Age (per year)	1.00 (0.98–1.02)	0.96
Length of Stay (per day)	0.92 (0.85–1.01)	0.08
DRG weight	1.20 (0.92–1.56)	0.17
Female Sex	1.28 (1.05–2.60)	0.02
Total Elixhauser Comorbidity Score	1.14 (0.95–1.36)	0.14
Education (compared with high school)		
Less than High School	0.75 (0.28–1.32)	0.35
Some College	0.79 (0.38–1.09)	0.24
College Graduate	1.57 (0.67–2.45)	0.06
Post-Graduate	0.87 (0.34–1.49)	0.63
Living Alone	1.08 (0.63–2.17)	0.61
Marital Status (compared with married or living as married)		
Divorced or separated	1.37 (0.75–3.15)	0.22
Widowed	0.90 (0.50–2.03)	0.70
Single never married	0.89 (0.52–1.96)	0.66
Rural Area of Residence	1.359	0.03
Insurance (compared with Private Insurance)		
Medicaid	0.86 (0.26–1.76)	0.70
Medicare	0.96 (0.42–1.35)	0.89
Self-insured	0.94 (0.21–2.62)	0.90
Coronary Artery Disease	1.11	0.38
Type 2 Diabetes Mellitus	1.22	0.06
Hypertension	1.20	0.21
Rural**Coronary Artery Disease	1.28	0.02
Rural**Type 2 Diabetes Mellitus	0.90	0.34
Rural**Hypertension	0.63	0.0005

*The multiple logistic regression model outcome indicates whether or not a patient experienced at least one adverse event.

**These risk factors in the model indicate the effect of hypertension, type 2 diabetes mellitus, and coronary artery disease with adverse event risk in rural patients.

DRG = Diagnosis Related Group.

## Discussion

In this study, we found that patients with planned follow-up visits within 7 days of discharge paradoxically had a higher incidence of post-discharge AEs than those whose follow-up was scheduled later. This effect persisted after adjustment for several patient factors aimed to reduce confounding by indication, including patient age, DRG weight of the primary diagnosis, insurance status, and several common comorbidities.

We expected that our results would indicate that patients who had scheduled post-discharge follow-up visits sooner would be less, not more, likely to experience a post-discharge AE. Our findings may indicate that hospitalist physicians were accurately identifying patients at discharge deemed to be at high risk for post-discharge problems and therefore scheduled faster follow-up with them (i.e., confounding by indication). This informal risk assessment by clinicians may have been based in part on medical complexity (which we were able to adjust for, at least in part) but also psychosocial complexity, differences in health literacy, perceived stability at discharge, or functional status, which we were not able to adjust for (although we were able to account for living alone and insurance status). It is notable that the effect of early follow-up was attenuated with these adjustments, consistent with our hypothesis, but did not disappear or reverse in direction, which might be expected if adjustment were complete. We cannot exclude the possibility that our findings also reflect information bias. Patients with faster follow-up may have had better documentation and patient awareness of symptoms that could be collected during the follow-up and outcome adjudication process. It is less likely that our results were due to reverse causality (i.e., faster follow-up due to an AE) because we restricted our analysis to follow-up scheduled at the time of discharge.

There have been several studies that have evaluated the association of timely post-hospital follow-up and hospital readmissions. The largest observational study of readmissions in Medicare patients found that most readmissions occurred before any scheduled follow-up, at least implying that follow-up should have occurred sooner [16]. A recent study found that it would be most beneficial to target high risk patients for early post-discharge follow-up within 7-days [1]. Retrospective studies tend to show no relationship between early follow-up (either within 7 or 14 days) and readmission rates, likely because of the same confounding by indication issues noted above. To our knowledge, this is the first study to examine the effect of early follow-up on post-discharge adverse events as opposed to readmission rates.

Our study in no way implies that early follow-up is actually harmful to patients. The results should serve as a word of warning to using observational studies to evaluate transitional care interventions, especially those reserved for a subset of patients, such as early follow-up, where the risks of confounding by indication are particularly high. The results also imply that the true effect of early follow-up (at least with respect to post-discharge follow-up) is essentially unknown, might be smaller than suspected, and needs studies with non-observational study designs to quantify accurately.

Our study had several limitations. As noted above, we were unable to capture several likely confounders of the relationship between timeliness of follow-up and post-discharge AEs, including several markers of real and perceived risk. We also were unable to collect data on the nature of the follow-up, e.g., what topics were discussed, how many components of ideal transitional care were implemented. Additionally, it was necessary to restrict the evaluation to patients with follow-up visits that had been scheduled prior to discharge to avoid reverse causality (i.e., adverse events causing follow-up visits). However, because patients with an unplanned visit may be the same as patients without (or with late) scheduled visits, by excluding them, we could mask the association between late (or no) follow-up and poor outcomes, i.e., the expected result and the opposite of what we found. Finally, in observational research, there is a risk that those perceived as sicker may be selected for more intensive interventions by their providers. Therefore, it may seem as if those receiving interventions have worse outcomes than those that do not receive the interventions, implying that the interventions are harmful, while in fact it is the baseline characteristics of those selected for the interventions that is the issue. Moreover, not all of these baseline characteristics can be measured, or even known, and therefore cannot be adjusted for in multivariable models. Indeed, this “confounding by indication” is the major finding of this study.

## Conclusion

Further research is needed to determine the relationship between early post-discharge follow-up visits and post-discharge AEs. This includes the perceived risk factors that influence the decision to schedule early follow-up and how well they match with actual risk, and the likelihood of benefitting from interventions. Further work is also needed to better understand the optimal timing and nature of follow-up in different sub-populations of high risk patients. Lastly, we continue to advocate for transitional care studies that evaluate not just readmissions but post-discharge AEs, which are very common, can have a profound effect on patients’ quality of life, and are likely more amenable to change than readmissions.

## Supporting information

S1 Table Telephone interview questionnaire. (DOC)

S2 Table A comparison of patient characteristics for patients included in the study (N = 545) and patients excluded from the study (N = 168 and N = 271). (DOCX)
