# Supplementary material for: The association of post-discharge adverse events with timely follow-up visits after hospital discharge
Source: PLoS One. 2017 Aug 10;12(8):e0182669. doi: 10.1371/journal.pone.0182669 (PMC5552135; doi:10.1371/journal.pone.0182669)
Supplement: S1 Table — (DOC) [file pone.0182669.s001.doc]

**S1 Table. Telephone Interview Questionnaire**

| 1. | Did the hospital staff take your preferences and those of your family or caregiver into account in deciding what your health care needs  would be when you left the hospital?  (a) Yes  (b) No |
| --- | --- |
| 2. | When you left the hospital, did you have a good understanding of the things you were responsible for in managing your health?  (a) Yes  (b) No |
| 3. | When you left the hospital, did you clearly understand the purpose for taking each of your medications?  (a) Yes  (b) No |

|  | Have you seen your doctor  since discharge?  1=Yes 2=No 3=NA | If yes, did your doctor know  of your hospitalization?  1=Yes 2=No 3=NA | If no, what is the date of  your next appointment  with your doctor?  MM/DD/YY | In your opinion, would an earlier appointment  have been helpful?  1=Yes 2=No 3=NA |
| --- | --- | --- | --- | --- |
| 4. | (a) | (b) | (c) | (d) |
| 5. | (a) | (b) | (c) | (d) |

| 6. | Have you received home care services (like a visiting nurse) since returning home?  (a) Yes  (b) No |
| --- | --- |
| 7. | If you answered no, do you feel you needed them?  (a) Yes  (b) No |
| 8. | Have you returned to the emergency department since your discharge from the hospital?  (a) Yes  (b) No |
| 9. | If so, why? |
| 10. | Did you require readmission to the hospital since your initial visit?  (a) Yes  (b) No |
| 11. | If so, why? |

|  |  | Symptom | How long  ago did this  symptom  start? | If it started before  discharge, has it  gotten worse  since getting  home? | Is this symptom related to your recent hospitalization? | Have you discussed it with your doctor? | Did your doctor do anything in response? | What did he/she do? | Did this symptom require an additional visit to a medical facility? |
| --- | --- | --- | --- | --- | --- | --- | --- | --- | --- |
|  |  | 1 = No  2 = Yes  3 = DK | 1 = After  discharge  2 = During  admission  3 = <1 month before admission  4 = >1 month  before admission | 1 = No  2 = Yes  3 = DK | 1 = No  2 = Yes  3 = DK | 1 = No  2 = Yes | 1 = No  2 = Yes | 1=Arrange visit  with him/herself  2=Change treatment over telephone  3=Arrange visit with other health provider  4=Arrange referral to emergency department | 1 = No  2 = Clinic visit  3 = Emergency department visit  4=Hospitalization  5=Other |
| 12. | Fever |  |  |  |  |  |  |  |  |
| 13. | Pain or discomfort  (specify location:  1=head 2=chest 3=abdomen 4=back 5=extremity) |  |  |  |  |  |  |  |  |
| 14. | Inability to eat |  |  |  |  |  |  |  |  |
| 15. | Nausea or vomiting |  |  |  |  |  |  |  |  |
| 16. | Diarrhea |  |  |  |  |  |  |  |  |
| 17. | Shortness of breath |  |  |  |  |  |  |  |  |
| 18. | Cough |  |  |  |  |  |  |  |  |
| 19. | Skin breakdown  (specify location:  1=back  2=lower extremity  3=other) |  |  |  |  |  |  |  |  |
| 20. | Rash |  |  |  |  |  |  |  |  |
| 21. | Falls |  |  |  |  |  |  |  |  |
| 22. | Swollen Legs |  |  |  |  |  |  |  |  |
| 23. | Urinary frequency |  |  |  |  |  |  |  |  |
| 24. | Dysuria |  |  |  |  |  |  |  |  |
| 25. | Other (please specify) |  |  |  |  |  |  |  |  |

DK = don’t know; NA = not available.
