# Supplementary material for: The association of post-discharge adverse events with timely follow-up visits after hospital discharge
Source: PLoS One. 2017 Aug 10;12(8):e0182669. doi: 10.1371/journal.pone.0182669 (PMC5552135; doi:10.1371/journal.pone.0182669)
Supplement: S2 Table — (DOCX) [file pone.0182669.s002.docx]

**S2 Table**: A comparison of patient characteristics for patients included in the study (N=545) and patients excluded from the study (N=168 and N=271).

| Number of sample | *N=545, (%) | **N=168, (%) | ***N=271, (%) |
| --- | --- | --- | --- |
| Age, mean (SD), range | 61.7 (14.74), 22-93 | 57.46 (17.14), 21-93 | 65.62 (16.39), 21-96 |
| Sex |  |  |  |
| Female | 278 (51.0) | 89 (53.0) | 126 (46.5) |
| Male | 267 (49.0) | 79 (47.0) | 145 (53.5) |
| Race |  |  |  |
| White | 428 (78.5) | 105 (62.5) | 193 (71.2) |
| Non-White | 117 (21.5) | 63 (37.5) | 78 (28.8) |
| Education |  |  |  |
| Less than high school | 56 (10.3) | 19 (11.3) | N/A |
| High school | 184 (33.8) | 63 (37.5) | N/A |
| Some college | 171 (31.4) | 42 (25.0) | N/A |
| College degree | 74 (13.6) | 33 (19.6) | N/A |
| Post-graduate | 60 (11.0) | 11 (6.5) | N/A |
| Household income |  |  |  |
| Less than $9,000 | 52 (10.9) | 36 (21.4) | N/A |
| $9000 - 24,999 | 111 (23.3) | 56 (33.3) | N/A |
| $25,000 – 49,999 | 120 (25.2) | 26 (15.5) | N/A |
| $50,000 – 74,999 | 97 (20.3) | 14 (8.3) | N/A |
| $75,000 – 99,999 | 56 11.7) | 10 (6.0) | N/A |
| $100,000 or more | 41 (8.6) | 8 (4.8) | N/A |
| Missing | 68 (12.5) | 18 (10.7) | N/A |
| Living situation |  |  |  |
| Lives alone | 93 (17.1) | 133 (79.2) | N/A |
| Does not live alone | 452 (82.9) | 35 (20.8) | N/A |
| Insurance type |  |  |  |
| Private | 204 (37.4) | 50 (29.8) | N/A |
| Medicare | 285 (52.3) | 76 (45.2) | N/A |
| Medicaid | 38 (7.0) | 22 (13.1) | N/A |
| Self-pay | 18 (3.3) | 20 (11.9) | N/A |
| Living location |  |  |  |
| Urban | 279 (51.2) | 79 (47.0) | 142 (52.4) |
| Rural | 266 (48.8) | 89 (53.0) | 129 (47.6) |

*The 545 patients were included in the study. **The 168 patients were excluded from the study because 29 of these patients did not have an interview nor health records available, 81 of these patients did not have post-discharge health records, and 58 of these patients had unplanned visits after hospital discharge. ***The 271 patients decline participation in the study and limited information was collected from these patients.
